# Personality traits predict brain activation and connectivity when witnessing a violent conflict

**DOI:** 10.1038/srep13779

**Published:** 2015-09-04

**Authors:** Jan Van den Stock, Ruud Hortensius, Charlotte Sinke, Rainer Goebel, Beatrice de Gelder

**Affiliations:** 1 Laboratory for Translational Neuropsychiatry, Department of Neurosciences, KU Leuven, Leuven, Belgium; 2 Old Age Psychiatry, University Hospitals Leuven, Herestraat 49, 3000 Leuven, Belgium; 3 Brain and Emotion Laboratory, Department of Cognitive Neuroscience, Faculty of Psychology and Neuroscience, Maastricht University, Oxfordlaan 55, 6200 MD Maastricht, the Netherlands; 4 Department of Psychiatry & Mental Health, University of Cape Town, J-Block, Groote Schuur Hospital, Cape Town, South Africa

## Abstract

As observers we excel in decoding the emotional signals telling us that a social interaction is turning violent. The neural substrate and its modulation by personality traits remain ill understood. We performed an fMRI experiment in which participants watched videos displaying a violent conflict between two people. Observers’ attention was directed to either the aggressor or the victim. Focusing on the aggressor (vs. focusing on the victim) activated the superior temporal sulcus (STS), extra-striate body area (EBA), occipital poles and centro-medial amygdala (CMA). Stronger instantaneous connectivity occurred between these and the EBA, insula, and the red nucleus. When focusing on the victim, basolateral amygdala (BLA) activation was related to trait empathy and showed increased connectivity with the insula and red nucleus. STS activation was associated with trait aggression and increased connectivity with the hypothalamus. The findings reveal that focusing on the aggressor of a violent conflict triggers more activation in categorical (EBA) and emotion (CMA, STS) areas. This is associated with increased instantaneous connectivity among emotion areas (CMA-insula) and between categorical and emotion (EBA-STS) areas. When the focus is on the victim, personality traits (aggression/empathy) modulate activity in emotion areas (respectively STS and postcentral gyrus/ BLA), along with connectivity in the emotional diencephalon (hypothalamus) and early visual areas (occipital pole).

Social interactions are very much a part of our daily life, whether or not we are actively involved in them. When observing interactions from a third person perspective we are adept at recognizing the relational^1^ and emotional^2^ dynamics at stake in dyadic interactions. There is evidence that observers selectively process emotion-cues contained in a complex scene^3^,^4^,^5^, but the matter is more complicated when observing an interaction between two people, as the focus of attention may be drawn to either of the two people involved. A sensible assumption is that the actual focus of attention influences eyewitness reports^6^. The present study examined this selective attention issue of the observer, but not about identifying a perpetrator. We presented realistic video clips of aggressive two-person interactions in which one agent was clearly the aggressor and the other was the victim. The video clips were identical across conditions, but the focus of attention (on aggressor or on victim) was manipulated. Participants performed a dot color discrimination task unrelated to the content of the videos but forcing them to attend to the location of either the aggressor or the victim. The use of an orthogonal task is useful because it allows one to investigate brain responses that are not influenced by the task but by the underlying differences of the variables of interest.

When observers see two people approach each other to shake hands, they typically focus more on one of both. However, this is not the case when observers see the same people facing away from each other^7^. However, there is currently no evidence documenting the specific neural correlates involved. We expected that focusing on the aggressor would give rise to more activation in areas related to processing threat, such as the amygdala and premotor cortex (PMC). In addition, activity in regions important for preparation for action, such as the supplemental motor area and middle frontal gyrus^8^,^9^ should facilitate focusing on the aggressor.

Secondly, when social interactions consist of more than a neutral exchange of information, the emotional involvement of the protagonists may prompt the observer to choose sides. However, there is substantial inter-individual heterogeneity in how people respond when they are present at the scene of a violent conflict^10^. Affiliative behavior with either the aggressor or the victim has been linked to trait empathy and this plays a key role in both emotional experience and social interactions. Empathy is associated with tendencies for prosocial behaviour and is particularly relevant during the observation of a violent conflict as observing another’s distress often leads to helping behaviour^11^. At the neural level, pain studies have revealed that pain felt by the subject as well as vicariously felt pain when observers witness painful stimulation of a loved one, activates overlapping regions in the anterior insula (aIns) and dorsal anterior cingulate cortex (dACC), areas that have been associated with subjective evaluation of pain, rather than with the objective intensity of the nociceptive stimulus^11^. We included a trait measure of empathy to investigate how it modulates brain activation when witnessing a violent conflict. Based on the findings reported above and the postulated function of the aIns in integrating interoceptive signals into a subjective affective experience^12^, we hypothesise that trait empathy will modulate activation in the aIns and dACC when the observer is focussing on the victim^11^. Furthermore, we included trait aggression as a personality variable, as witnessing aggression is often a trigger for aggressive acts. In addition, there is evidence documenting inter-individual differences in how observers respond to a violent conflict. For instance, while some react with aggression, others respond submissively^10^. Aggression has been related to decreased response inhibition and higher motor impulsivity and has, at the neural level, been associated with hypo-activation of motor and somatosensory cortices, including during perception of angry emotions^13^,^14^. Based on these findings, we hypothesize aggression-related modulation of motor- and somatosensory areas.

Finally, we follow-up on the observed neural activations by computing their underlying functional connectivity. This will allow us to investigate how differential neural activations are associated with (sub- or supra-threshold) differential activations in distant areas. This reveals which areas communicate more with the reference region during a particular task^15^. Specifically, we investigate the connectivity associated with neural activations when the focus is on the aggressor compared to when the focus is on the victim. We hypothesize differential connectivity with areas related to categorical body perception such as the extra-striate body area (EBA)^16^ and fusiform body area (FBA)^17^, as well as with areas associated with body emotion processing such as the amygdala, insula and premotor cortex^18^.

## Results

One subject was excluded from analysis because of excessive movements. The resulting N for all tests reported below therefore equaled 14. All tests are two-tailed.

### Behavioral results

Reaction times were processed using the hybrid moving criterion method^19^ and an R-script developed by Grange^20^. This procedure allows for the removal of outliers and takes into account the number of trials per condition and adapts the cut-off value accordingly. Mean percentage of trials removed was 1.83 (SD = 0.84). For the Aggression Questionnaire measure, we used the total score.

Accuracy was well above chance level (mean = 81.3%; SD = 3.7) as measured by a one-sample t-test (t(13) = 85.51; p < .001). There were no significant differences in accuracy (t(13) = 0.38; p = .71), nor reaction times (t(13) = 0.76; p = .46) as a function of attentional focus (i.e. on the aggressor vs victim), as revealed by paired-sample t-tests.

**Figure 1:**
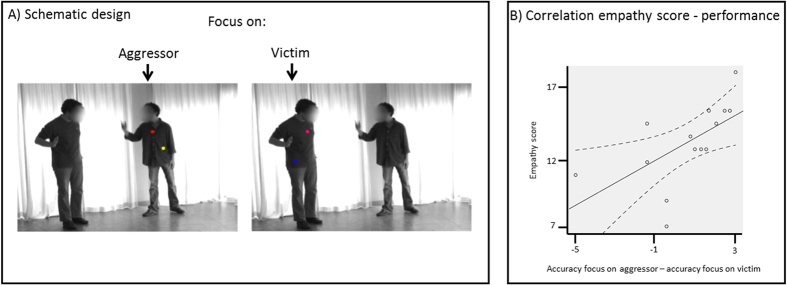
(**A**) Schematic presentation of design and stimuli. Videos of a violent conflict between two individuals were presented while participants were instructed to ignore the visual scene and to indicate whether two consecutive dots were of the same color or not. The location of the dots (on the left or the right person) was signaled prior to the onset of the stimuli. For illustrative purposes, both dots are displayed here together. (**B**) Scatterplot display showing the correlation between the score on the empathy scale and the difference between the accuracy when the focus was on the aggressor compared to when the focus was on the victim. The full line represents the linear fit, while the dashed lines represent the 95% mean confidence range.

Means, standard deviations and range of Aggression and Empathy scores are presented in Supplementary Table S1. We computed Spearman Correlation Coefficients of Aggression and of Empathy scores with the behavioral performance (accuracy and reaction times). The questionnaire scores were correlated with each condition separately as well as with the difference between both conditions. This revealed a significant negative correlation between the Aggression-score and reaction time when the focus was on the aggressor (ρ(14) = −.556, *p* = .039). The Empathy score correlated positively with the accuracy difference when the focus was on the aggressor minus on the victim (ρ(14) = .77, *p* = .001) (see Fig. 1B).

### Imaging results

fMRI data were analyzed using BrainVoyager QX (version 2.8, Brain Innovation, Maastricht, the Netherlands)^21^. The first two volumes of every run were excluded from the analysis to permit T1 equilibration effects. Preprocessing included slice scan time correction by means of cubic spline interpolation, 3D motion correction by means of trilinear/sinc interpolation, temporal high-pass filtering (GLM-Fourier) of 2 sines/cosines and spatial smoothing with a Gaussian filter of 8mm FWHM in the space domain. Functional data were co-registered with the anatomical volume and transferred into Talairach space. The statistical analysis was based on the general linear model (GLM), with each condition defined as a predictor plus one for the instruction screen. Cortical results were analyzed in surface space, while non-cortical results were analysed in subcortical space as defined by a commonly used atlas^22^, which was adapted for TAL-space. Cluster-size correction was used with an initial threshold of *p* = .01, and 1000 Monte Carlo simulations, unless stated otherwise.

To relate the results from the interaction experiment to category selective areas, we first analyzed the localizer data and defined: the extra-striate body area (EBA)^16^ (bodies vs. houses and tools), fusiform body area (FBA)^17^ (bodies vs. houses and tools) and parahippocampal place area (PPA)^23^ (houses vs. bodies, faces and tools). In addition, we projected the frontal eye field (FEF), the hand motor area (M1_hand) and hand somatosensory area (S1_hand) based on a probabilistic atlas^24^. The plotted regions were defined by the areas showing overlap in >20% of the subjects.

**Figure 2:**
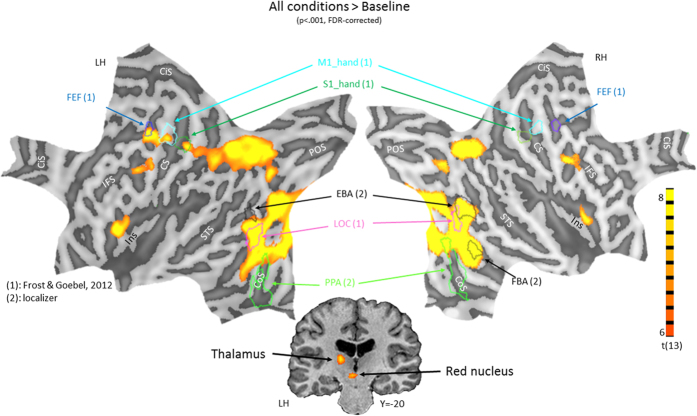
Results of the contrast of all conditions vs baseline. Statistical maps of activation are displayed on flattened surfaces of the left (left) and right (right) hemisphere. Gyri are shown in light grey and sulci in dark grey. Outlines of relevant regions of interest are projected in the surfaces and consist of the frontal eye fields (FEF), primary hand-motor area (M1_hand), primary hand somatosensory area (S1_hand), extra-striate body area (EBA), lateral occipital complex (LOC), parahippocampal place area (PPA) and fusiform body area (FBA). In addition, anatomical gyral and sulcal landmarks are indicated in black and white respectively and consist of the cingulate sulcus (CiS), inferior frontal sulcus (IFS), central sulcus (CS), superior temporal sulcus (STS), collateral sulcus (CoS), parieto-occipital sulcus (POS) and insula (Ins). The central bottom figure displays subcortical activations in the thalamus and red nucleus. Y refers to Talairach coordinate.

Next, we investigated the neural correlates of perceiving emotional two person interactions by comparing all conditions with the baseline (p < .001, FDR-corrected). The baseline was explicitly defined by the fixation blocks that alternated with the stimulation blocks. The results are displayed in Fig. 2 and Supplementary Table S2 and include bilateral anterior insula, premotor cortex, visual cortex, intraparietal sulcus (IPS), left precentral sulcus, precentral gyrus, postcentral gyrus, thalamic ventral postero-lateral nucleus and red nucleus. The left pre- and postcentral gyrus activations fall within the primary hand-motor and somatosensory regions respectively, as the outlines in Fig. 2 reflect. This suggests that these activations are most likely related to button press responses. In addition, the left FEF were activated during observation of two-person interactions. The activation in the visual cortex showed extensive overlap with the right EBA, FBA and bilateral LOC, but little with the PPA. There were no significant correlations at whole brain level with empathy or aggression scores.

**Figure 3:**
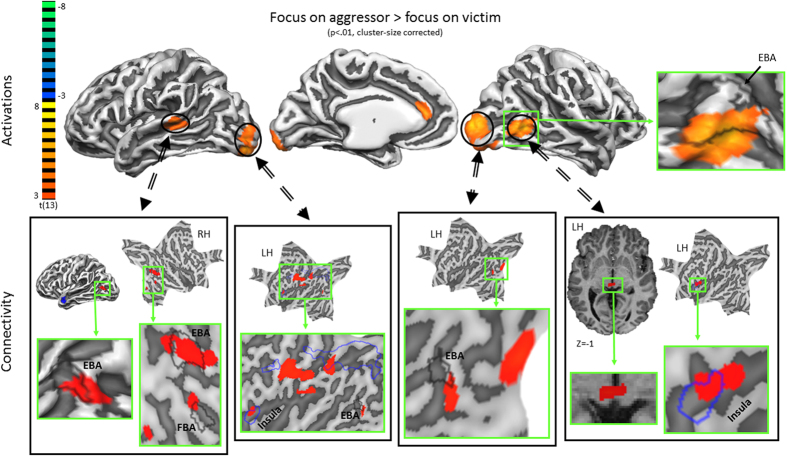
Results of the contrast focus on aggressor vs. focus on victim showing statistical activation maps (top row) and the associated differential connectivity patterns (bottom row) of the activation clusters. Body-selective areas (EBA and FBA) are outlined in black, while the areas that were activated for the contrast all conditions vs baseline are outlined in blue.

**Figure 4:**
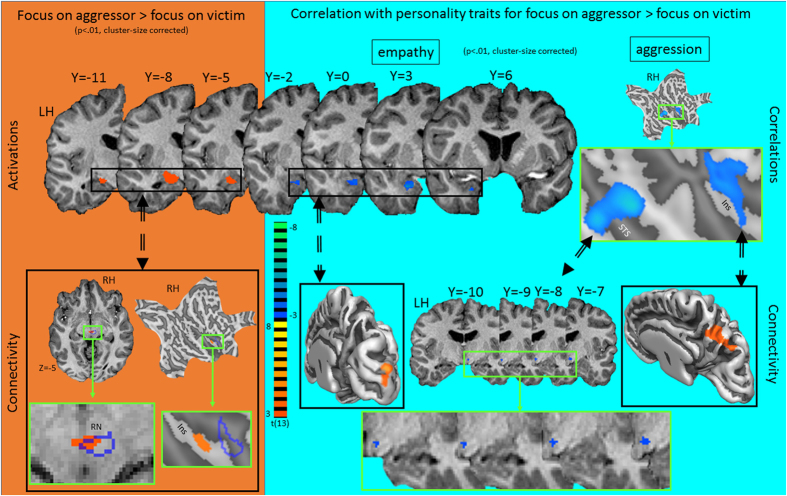
Results of the contrast focus on aggressor vs. focus on victim (against orange background) and correlations with personality traits empathy and aggression (against cyan background) showing statistical activation and correlation maps (top) and the associated differential connectivity patterns (bottom) of the clusters. Outlines of relevant regions of interest are projected based on a probabilistic atlas^24^ and consist of the frontal eye fields (FEF), primary hand motor area (M1_hand) and primary hand somatosensory area (S1_hand), In addition, areas that were activated for the contrast-all conditions vs. baseline are outlined in blue (RN: red nucleus) and anatomical gyral and sulcal landmarks are indicated in black and white respectively and consist of the central sulcus (CS) and insula (Ins). Z and Y refer to Talairach coordinates.

Secondly, we investigated the effect of attentional focus by comparing the condition with the focus on the aggressor vs. that with the focus on the victim. The results revealed heightened activation in the left anterior cingulate cortex (ACC), superior temporal sulcus (STS), amygdala, right middle occipital gyrus and bilateral occipital pole when the focus was on the aggressor (Figs 3 and 4, Table S2). Activation in the left amygdala correlated negatively with empathy-score and activation in the right postcentral gyrus and STS correlated negatively with the score on the aggression subscale. There were no significant results for the inverse contrast (i.e. focus on victim vs. focus on aggressor). There was no overlap between the two clusters in the amygdala. In line with reports documenting different specific functions to amygdalar subregions^25^, we related the amygdalar clusters to the results of a citoarchitectonic parcellation study^26^. This revealed that the activation related to the focus on the aggressor was primarily situated in the centro-medial part (CMA), while the empathy related activation was primarily located in the basolateral part (BLA).

We examined connectivity patterns associated with the respective activation and correlation clusters. For this purpose, we first defined new regions of interest (ROIs), consisting of the 300 most active voxels of the regions that were obtained in the focus on aggressor vs. focus on victim contrast, or of the regions that were modulated by trait empathy or aggression. We restricted the size of these reference regions to minimize the loss of temporal detail. Subsequently, we computed two voxel-wise instantaneous correlation maps of these regions. The first map contained the temporal correlations during the focus on aggressor condition, while the second contained the correlations during the focus on victim condition. Subsequently, we subtracted these two correlation maps at the subject level. Finally, for the trait-modulated regions, this difference was correlated with the respective trait score, while for the other regions the group average of the difference was tested against zero by means of a one-sample t-test.

The results are presented in Figs 3 and 4 and Table S2. The left STS and occipital poles showed stronger connectivity with areas that overlapped with the EBA. The activated cluster that overlapped with the EBA showed stronger connectivity with the insula and superior colliculi. The CMA cluster showed stronger connectivity with the right insula and left red nucleus (falling partly within the cluster that responded to perceiving emotional dyadic interactions), while the BLA showed stronger connectivity with the occipital pole. Finally, the S1 correlation cluster showed stronger connectivity with the superior parietal lobule (SPL) and ths STS with the right hypothalamus.

## Discussion

We investigated the neural basis of witnessing a violent conflict using video clips of two individuals engaged in an aggressive interaction and by varying the focus of attention on the aggressor or the victim. The results reveal activation of a distributed set of regions that have also been associated with body and emotion perception, including the insula, PMC, EBA and FBA, consistent with previous studies on social interactions^2^,^7^,^27^,^28^,^29^,^30^,^31^,^32^.

In addition, we observed activation in subcortical structures, specifically in thalamus and red nucleus. The red nucleus (nucleus ruber) is situated in the midbrain adjacent to the substantia nigra and is somatotopically organized. It receives somatotopical cortico-rubral projections from primary, pre-, supplementary and cingular motor cortex, frontal and supplementary eye fields, as well as cerebellar afferents. It projects to the olivo-cerebellar system via the inferior olive and to a lesser extent (in humans) to the spinal cord via the rubrospinal tract^33^. There is only limited empirical evidence so far on the function of the red nucleus however, it may play a more important role than envisaged hitherto. Interestingly, its implication in emotion processing, and more specifically, in defensive behavior^33^ as well as the subjective experience of affect (i.e. ‘feelings’), has been postulated^34^. The present results provide evidence that this structure is involved in perceiving socio-emotional cues and the connectivity results (discussed further below) indicate a functional coupling with the CMA. However, without a matched non-emotional condition, it is difficult to say whether the activation of the red nucleus was associated with perceiving socio-emotional cues, or whether it is simply involved in interpersonal perception or any number of other perceptual or decision-making processes.

The first objective of the study was to investigate the neural activations of the focus on either the aggressor or the victim during observation of a dyadic interaction. The results revealed that focusing on the aggressor triggered more activation in the AMG, EBA, ACC, STS and occipital pole, while there were no regions that showed a higher response when the focus was on the victim. Considering that the aggressor expressed anger whereas the victim expressed fear, these results are in line with a previous study in which perception of (solitary) angry body language was compared with perception of (solitary) fearful body language^35^, which indeed showed increased activation for anger vs fearful body perception in the occipital pole, STS, EBA and ACC. Furthermore, the present results suggest that neural processing of emotional interactions is strongly dependent on the emotional cues conveyed by the agent at the center of attention. This asymmetric contribution emerges not only when the activations are directly compared, but is also reflected in their underlying connectivity. Moreover, the connectivity results of the EBA itself include superior colliculi and insula. Both these regions, as well as the EBA, have been implicated in automatic processing of bodies and body expressions, as clinically demonstrated by studies with cortically blind patients^36^,^37^. These findings add to the accumulating evidence that the function of EBA goes beyond the mere conscious perception of body shape and extends to visual emotion cues^5^,^38^, context effects^4^, motor actions^39^, crowd perception^32^ or non-conscious body processing^37^,^40^.

These results are consistent with findings from a recent study, which also used realistic interaction videos. The videos showed either an aggressive interaction, in which one agent was trying to grab the purse of the other, or a playful interaction, in which one agent was teasing the other by playfully pulling her purse. The results revealed that the AMG was more activated during the threatening than during teasing interactions, together with areas associated with action preparation (PMC, putamen). Based on these results, it could be argued that witnessing a violent conflict results in increased action readiness^2^,^18^ although alternative explanations, for instance based on action or movement perception, cannot be ruled out^41^,^42^,^43^,^44^.

In addition, when the focus was on the aggressor, the CMA was more active and also showed stronger connectivity with the red nucleus and insula. There is evidence that the CMA is the primary output region of the AMG and projects to cortical and subcortical areas, including the insula and brainstem^45^,^46^. Furthermore, a recent study investigated effective connectivity during observation of martial arts and reported output from CMA to brainstem and insula^47^. Against this background, we hypothesize that the increased functional coupling we observe here between CMA on the one hand and the red nucleus and insula on the other, reflects increased output from the CMA when the focus is on the aggressor, possibly indicating increased emotion generation and awareness respectively^34^.

The second main objective of this study was to investigate how empathy and aggression traits influence the neural and behavioral processing of witnessing a violent conflict. The behavioral task was orthogonal to the research question and consisted of color discrimination of dots presented on either the aggressor or the victim. The results reveal that participants with a higher trait aggression were faster to respond when the focus was on the aggressor. The empathy score, however, was significantly related to how the expression of the (task irrelevant) background agent influenced behavioral performance. Participants with the lowest empathy scores were more accurate when the dots were presented on the victim, whereas the opposite pattern was observed for participants with the highest empathy scores. This may indicate that the more empathic subjects were more distracted when they focused on the aggressor, i.e. the angry body language, as previously documented^48^, whereas the attention of the less empathic subjects was primarily distracted (towards the aggressor) when they were focusing on the victim. We hypothesize that the attention of the more empathic participants was more drawn to the victim, as empathy has been related to prosocial behavior^11^. This behavioral observation was also reflected at the neural level. Participants with higher empathy scores displayed more pronounced differences in BLA activity according to whether they were focusing on the aggressor or on the victim. Functionally, the BLA has been associated with automatic filtering of irrelevant emotional cues. This has been documented most clearly in humans with bilateral damage to the BLA. These patients show increased sensitivity to task irrelevant emotional face and body expressions^49^,^50^. Regarding connectivity, the BLA is primarily associated with receiving and sending projections to and from cortical areas, including V1^51^. Our findings add to these reports by revealing that the connectivity (focus on aggressor vs victim) of the BLA with the occipital pole is modulated by trait empathy. This finding also extends previous results^47^ which included modulation of personality traits (psychopathy) of connectivity between BLA and occipital regions.

Furthermore, we observed a negative correlation between focus on the aggressor and trait aggression in the right STS and insula. While these have typically been associated with perception of emotional expressions^52^, feelings and interoception^12^, they have also been related to aggression^53^,^54^. Furthermore, the STS correlation was related to connectivity with the hypothalamus, whose involvement in aggression has been well documented^55^.

To conclude, the findings reveal that when witnessing a violent conflict, focusing on the aggressor compared to on the victim activates categorical (EBA) and emotion (CMA, STS) areas. This is associated with increased instantaneous connectivity among emotion areas (CMA-insula) and between categorical and emotion (EBA-STS) areas. When the focus is on the victim, personality traits (aggression and /empathy) modulate activity in emotion areas (respectively STS and S1/BLA), along with connectivity in the emotional diencephalon (hypothalamus) and early visual areas (occipital pole).

## Methods

The study was carried out in accordance with the Declaration of Helsinki. All experimental protocols were approved by the Ethical Committee Psychology (ECP) of Maastricht University.

### Participants

Fifteen healthy right-handed volunteers (all male; age 23.6 ± 4.1) participated in the study after providing written informed consent. All participants had normal or corrected-to-normal vision. Based on earlier findings showing the strongest neural effects when males observe emotional expressions displayed by other males, only male actors and male participants participated in order to minimize the impact of gender and hormonal fluctuations^56^.

### Materials

In order to create a controlled and realistic version of violent conflict, two professional actors were instructed to engage in an aggressive conversation and were videotaped. One of the actors (aggressor) was instructed to lash out at the other (victim). The latter was instructed to react in a defensive and submissive manner. To counterbalance the role of personal appearance and acting style, each actor played the aggressor in half of the videos and the victim in the other half.

The raw footage was edited into 1.5s grayscale movies (720 × 576 pixels; 11 × 16.5 cm on screen; 25 frames/second; 780 kbps data rate; 24 bit sample size) using Ulead VideoStudio (Version 10, Ulead Systems, Taipei, Taiwan) and further processed with Adobe After Effects (Version 6, Adobe Systems Inc, San Jose, CA, USA). The sound track was removed and faces were blurred. Next, all movies were validated regarding threat expression and realism. The video clips were presented one by one to observers (N = 9) who were instructed to indicate on a visual analogous scale (VAS) how threatening and how realistic the video clip appeared to them. Based on these results, 36 video clips (12 for each of the three actor-pairs) were selected as the most threatening and most realistic.

In order to create an orthogonal task with these materials, every video was edited by adding colored dots (40 ms each, visual angel = 0.26°) above the knees on one of the two individuals see also refs 9, 57. The two dots were pseudo-randomly presented (between dot interval; M = 728.06, SD = 197.13) with the first dot appearing in the first 1080 ms of the video clip (M = 531.94 ms, SD = 198.96 ms) and the second dot within the 1160–1360 ms time window (M = 1260, SD = 68.43). Eight different colors were used for the dots (red, pink, purple, blue, turquoise, green, yellow, and orange) and the colors in each movie could be the same or different. In a pilot study, these videos were presented to a different group of participants (N = 16) who were instructed to indicate whether the two dots were of the same color or not. The resulting accuracy was well above chance level, but showed no ceiling effect (M = 82.3; SD = 7.6). All colors appeared equally often in all conditions. Each of the 36 initially selected videos resulted in four new stimuli: one with similarly colored dots on the aggressor, one with similarly colored dots on the defender, one with differently colored dots on the aggressor and one with differently colored dots on the defender. In addition, all 144 (36 x 4) edited videos were mirrored along the vertical axis, resulting in a total of 288 unique stimuli. An example of a frame from a video with the dots included is shown in Fig. 1.

### Design and procedure

We used a blocked fMRI design with two conditions, one was with the focus on the aggressor and the other on the victim. The experiment consisted of four runs, each containing 17 fixation blocks of 14s, separated by 16 stimulus blocks of 24s. In one stimulus block, nine stimuli were presented with an inter-stimulus interval (blank screen) of 1167 ms. Each stimulus block was preceded by an instruction screen of 1500 ms showing a circle at the left or right side of the screen. This indicated whether the dots would appear on the person on the left or on the right in the interaction. Participants were instructed to ignore what happened in the interaction and indicate as quickly and accurately as possible by a button press whether the color of the second dot was the same or different from the first (buttons randomly reversed over participants). They had a practice run outside the scanner before the beginning of the session to become familiar with the procedure. The original and the mirrored movies were shown in separate runs. A total of 576 trials were presented, with 288 per condition.

At the end of the scanning session, an object category localizer was performed for the perception of faces, bodies, houses and tools. This included five 12s blocks of each stimulus category, interleaved with 14s fixation blocks. In one stimulus block twelve stimuli were presented for 450 ms, with an inter stimulus interval of 600 ms, and subjects were requested to perform a one-back task for details, see ref. 4.

Stimuli were displayed using Presentation software (Neurobehavioral Systems, Inc, version 11.0) on a screen located at the end of the scanner bore (at the side of the participants’ head) with a liquid crystal display (LCD) projector (PLC-XT11-16, Sanyo North America Corporation, San Diego, USA). The participants viewed the stimuli via a mirror mounted to the head coil at an angle of ±45°.

### fMRI data acquisition

The MRI unit used was a commercial head scanner with a magnetic field strength of 3T (Siemens Allegra, AG, Erlangen, Germany) and an eight-channel head coil.

The scan parameters for the functional run were: TR = 2000 ms, TE = 30ms, FA = 90°, base resolution = 64, FOV = 224 × 224 mm^2^, 32 interleaved slices of 3.5 mm (no gap), number of volumes = 326 per run (scan time = 10 m and 52s). Total scan time per session was about one hour. After the second functional run, a three-dimensional T1-weighted data set was scanned using parameters from the Alzheimer’s Disease Neuroimaging Initiative (ADNI), encompassing the whole brain (scan parameters: repetition time (TR) = 2250 ms, echo time (TE) = 26 ms, FA = 90°, FOV = 256 × 256 mm^2^, matrix size = 256 × 256, number of slices = 192, slice thickness = 1 mm, no gap, total scan time = 8 m and 26s).

The scan parameters for the localizer run were: TR = 2000 ms, TE = 30 ms, FA = 90°, base resolution = 112, FOV = 224 × 224 mm^2^, 28 interleaved slices of 2 mm (no gap), number of volumes = 267 (scan time = 8 m and 54s).

### Questionnaires

Following the scanning session, all participants completed a validated Dutch translation of the I7-questionnaire, developed by Eysenck and colleaguesas a measure of self-reported empathy^58^. The I7 questionnaire consists of 54 dichotomous items and includes three scales: impulsiveness, venturesomeness, and empathy. The empathy scale has been shown to primarily measure affective empathy, rather than cognitive empathy^59^. In addition, all participants completed a validated Dutch translation of the Aggression Questionnaire^60^ in which they rate 29 items using a five-point Likert scale. It includes four separate subscales (Hostility, Anger, Verbal Aggression and Physical Aggression).

## Additional Information

**How to cite this article**: Stock, J. V. d. *et al.* Personality traits predict brain activation and connectivity when witnessing a violent conflict. *Sci. Rep.* **5**, 13779; doi: 10.1038/srep13779 (2015).

## Supplementary Material

Supplementary Information
