# Supplementary material for: Personality traits predict brain activation and connectivity when witnessing a violent conflict
Source: Sci Rep. 2015 Sep 4;5:13779. doi: 10.1038/srep13779 (PMC4559660; doi:10.1038/srep13779)
Supplement: Supplementary Information [file srep13779-s1.doc]

Supplementary information:

Personality traits predict brain activation and connectivity when witnessing a violent conflict

Jan Van den Stock, Ruud Hortensius, Charlotte Sinke, Rainer Goebel & Beatrice de Gelder

Table S1. Empathy and Aggression scale details.

|  | Reliability | Mean | SD | Range |
| --- | --- | --- | --- | --- |
| Empathy | KR-20 = .67^1^ | 13.50 | 3.11 | 7-19 |
| Aggression | Cronbach’s α = .84^2^ | 67.07 | 21.07 | 48-110 |

Table S2. Imaging results.

|  | Peak activations | | | | | | | | |  |  |  |  |  |  |  |  |  |  |
| --- | --- | --- | --- | --- | --- | --- | --- | --- | --- | --- | --- | --- | --- | --- | --- | --- | --- | --- | --- |
|  |  |  |  | Nvoxels  (vertices) | X | Y | Z | t(r) | p |  |  |  |  |  |  |  |  |  |  |
| all conditions vs baseline |  |  |  |  |  |  |  |  |  |  |  |  |  |  |  |  |  |  |  |
|  | Red Nucleus |  | LH | 221 | -6 | -19 | -8 | 6.615 | .00001 |  |  |  |  |  |  |  |  |  |  |
|  | Thalamus |  | LH | 1919 | -18 | -25 | 4 | 8.959 | .000001 |  |  |  |  |  |  |  |  |  |  |
|  | OT |  | LH |  | -41 | -72 | -3 | 16.943 | .0000001 |  |  |  |  |  |  |  |  |  |  |
|  |  |  | RH | (1688) | 44 | -68 | -2 | 28.535 | .0000001 |  |  |  |  |  |  |  |  |  |  |
|  | IPS |  | LH |  | -34 | -50 | 49 | 11.566 | .0000001 |  |  |  |  |  |  |  |  |  |  |
|  |  |  | RH | (558) | 30 | -54 | 39 | 15.514 | .0000001 |  |  |  |  |  |  |  |  |  |  |
|  | Insula |  | LH |  | -27 | 22 | 7 | 8.342 | .000001 |  |  |  |  |  |  |  |  |  |  |
|  |  |  | RH | (121) | 30 | 19 | 6 | 7.285 | .000006 |  |  |  |  |  |  |  |  |  |  |
|  | preCS | dorsal (FEF/M1_hand) | LH |  | -28 | -14 | 49 | 8.981 | .000001 |  |  |  |  |  |  |  |  |  |  |
|  |  | ventral (premotor | LH |  | -39 | -1 | 30 | 6.892 | .00001 |  |  |  |  |  |  |  |  |  |  |
|  |  |  | RH | (173) | 42 | -1 | 32 | 9.300 | .0000001 |  |  |  |  |  |  |  |  |  |  |
|  | TOS |  | RH | (101) | 27 | -66 | 27 | 7.535 | .000004 | Peak connectivity | | | | | | | | | |
| focus Aggressor>victim |  |  |  |  |  |  |  |  |  |  |  |  |  | Nvoxels  (vertices) | X | Y | Z | t(r) | p |
|  | AMG | CMA | LH | (338) | -21 | -4 | -14 | 3.601 | .003 |  | Red Nucleus |  | LH | 11 | -3 | -19 | -5 | 3.218 | .006 |
|  |  |  |  |  |  |  |  |  |  |  | Insula |  | RH | (35) | 36 | 2 | 4 | 3.052 | .009 |
|  | ACC |  | LH |  | -8 | 34 | 16 | 4.335 | .0008 |  |  |  |  |  |  |  |  |  |  |
|  | OP |  | LH |  | -22 | -89 | -12 | 5.332 | .0001 |  | Insula |  | LH | (29) | -31 | 22 | 13 | 3.838 | .002 |
|  |  |  |  |  |  |  |  |  |  |  | MOG | EBA | LH | (72) | -47 | -73 | 5 | 3.053 | .009 |
|  |  |  |  |  |  |  |  |  |  |  | IPS |  | LH | (251) | -48 | -37 | 43 | 3.743 | .002 |
|  |  |  |  |  |  |  |  |  |  |  | Parietal Operculum | SII | LH | (121) | -48 | -33 | 25 | 3.322 | .005 |
|  |  |  |  |  |  |  |  |  |  |  | CS/postCG |  | LH | (742) | -48 | -10 | 28 | 4.035 | .001 |
|  |  |  | RH | (342) | 22 | -94 | 4 | 6.220 | .00003 |  | OP |  | LH | (53) | -18 | -101 | 2 | 4.530 | .0005 |
|  |  |  |  |  |  |  |  |  |  |  | CaS |  | RH | (44) | 11 | -91 | 1 | 5.219 | .0001 |
|  |  |  |  |  |  |  |  |  |  |  | MOG | EBA | LH | (83) | -47 | -73 | 5 | 3.639 | .003 |
|  | STS |  | LH |  | -57 | -40 | 10 | 4.249 | .0009 |  | MOG | EBA | LH | (41) | -47 | -73 | 8 | 3.074 | .008 |
|  |  |  |  |  |  |  |  |  |  |  |  | EBA | RH |  | 51 | -52 | 4 | 3.841 | .002 |
|  |  |  |  |  |  |  |  |  |  |  |  | TP | LH | (68) | -44 | 2 | -17 | -4.093 | .001 |
|  |  |  |  |  |  |  |  |  |  |  | FG | FBA | RH |  | 38 | -58 | -9 | 3.364 | .005 |
|  |  |  |  |  |  |  |  |  |  |  | IOG |  | RH |  | 9 | -73 | -5 | 3.488 | .004 |
|  | MOG | EBA | RH | (229) | 43 | -62 | 1 | 6.235 | .00003 |  | SC |  | LH/RH | 224 | -3 | -25 | 1 | 4.860 | .0003 |
|  |  |  |  |  |  |  |  |  |  |  | Insula |  | LH | (181) | -36 | 23 | 16 | 3.408 | .004 |
|  |  |  |  |  |  |  |  |  |  |  |  |  |  |  |  |  |  |  |  |
| r(trait Aggression) | Insula |  | RH | (422) | 34 | -4 | 15 | (-.704) | .004 |  | SPL |  | RH | (420) | 8 | -67 | 45 | 3.565 | .003 |
|  | STS |  | RH | (427) | 57 | -26 | 2 | (-.797) | .0006 |  | Hypothalamus |  | RH | 37 | 6 | -7 | -5 | 3.754 | .002 |
| r(trait Empathy) | AMG | BLA | LH | (294) | -24 | 5 | -20 | (-.730) | .003 |  | OP |  | RH |  | 18 | -94 | 11 | (.696) | .005 |

X,Y and Z refer to Talairach coordinates. OT=occipito-temporal cortex; IPS=intraparietal sulcus; PreCS=precentral sulcus; TOS=transverse occipital sulcus; AMG=Amygdala; CMA=centromedial amygdala; ACC=anterior cingulate cortex; OP=occipital pole; MOG=middle occipital gyrus; EBA=extrastriate body area; CS=central sulcus; CaS=calcarine sulcus; STS=superior temporal sulcus; TP=temporal pole; FG=fusiform gyrus; FBA=fusiform body area; IOG=inferior occipital gyrus; SC=superior colliculus; SPL=superior parietal lobule; BLA=basolateral amygdala.

1 Lijffijt, M., Caci, H. & Kenemans, J. L. Validation of the Dutch translation of the I7 questionnaire. *Personality and Individual Differences* **38**, 1123-1133 (2005).

2 Meesters, C., Muris, P., Bosma, H., Schouten, E. & Beuving, S. Psychometric evaluation of the Dutch version of the Aggression Questionnaire. *Behaviour research and therapy* **34**, 839-843 (1996).
